# Review of the Application of Dual Drug Delivery Nanotheranostic Agents in the Diagnosis and Treatment of Liver Cancer

**DOI:** 10.3390/molecules28207004

**Published:** 2023-10-10

**Authors:** Qinghe Han, Lianze Du, Lili Zhu, Duo Yu

**Affiliations:** 1 Radiology Department, The Second Affiliated Hospital of Jilin University, Changchun 130062, China; hanqinghe@jlu.edu.cn (Q.H.); dulianze0416@outlook.com (L.D.); irenezhu0810@outlook.com (L.Z.); 2 Department of Radiotherapy, The Second Affiliated Hospital of Jilin University, Changchun 130062, China

**Keywords:** liver cancer, dual drug delivery, anoparticles, diagnostic and therapeutic agents

## Abstract

Liver cancer has high incidence and mortality rates and its treatment generally requires the use of a combination treatment strategy. Therefore, the early detection and diagnosis of liver cancer is crucial to achieving the best treatment effect. In addition, it is imperative to explore multimodal combination therapy for liver cancer treatment and the synergistic effect of two liver cancer treatment drugs while preventing drug resistance and drug side effects to maximize the achievable therapeutic effect. Gold nanoparticles are used widely in applications related to optical imaging, CT imaging, MRI imaging, biomarkers, targeted drug therapy, etc., and serve as an advanced platform for integrated application in the nano-diagnosis and treatment of diseases. Dual-drug-delivery nano-diagnostic and therapeutic agents have drawn great interest in current times. Therefore, the present report aims to review the effectiveness of dual-drug-delivery nano-diagnostic and therapeutic agents in the field of anti-tumor therapy from the particular perspective of liver cancer diagnosis and treatment.

## 1. Introduction

Liver cancer is a common primary malignant tumor of the liver with relatively high incidence and mortality rates, and single treatment methods usually lead to poor outcomes [1]. The early detection and diagnosis of the occurrence and development of liver cancer, followed by the timely implementation of a multimodal combined treatment modality, remains challenging to date. Nanotechnology and its application in the field of medicine and human health have, fortunately, raised the hope of achieving an integrated diagnosis and treatment of liver cancer [2,3].

Synergistic treatment involves using two chemotherapeutic agents or a multimodal approach to treat a condition. When applied to the treatment of tumors, synergistic treatment could prevent the issues of drug resistance and side effects while reducing the possibility of tumor recurrence arising commonly due to the traditionally administered high doses of single drugs, thereby achieving a more desirable therapeutic effect [4,5]. In addition, the combined administration of drugs with different physicochemical properties and different mechanisms of action leads to better therapeutic outcomes. In particular, the order of administration of drugs is important when the drugs are capable of causing considerably different effects, synergistic or even opposite [6,7].

Recent studies have reported various nanoplatforms, such as liposomes [8], dendritic polymers [9], polymer nanoparticles (NPs) [10], and silica nanoparticles [11,12], with the capability to encapsulate a collection of different drugs and deliver them together to target (usually diseased) cells, thereby eliminating the side effects occurring due to the application of the respective drugs individually. Inorganic Janus NPs (JNPs) are capable of carrying different concentrations of drugs in their different compartments, without the interference of any of the drugs with the remaining ones. This is achieved because of the heterogeneous structure, surface properties, and various functions of JNPs and it greatly facilitates the independent release of individual drugs compared to that with the use of conventional nanoparticles with limited capacity to control the order of release of the drugs. Alternatively, two distinct compartments may be designed to synergize stimulus–response release properties (pH stimulation or NIR stimulation responsiveness) [13,14]. Inorganic JNPs exhibit great potential for application in dual-drug delivery systems. Therefore, the progress of research on the dual drug delivery systems for liver cancer diagnosis and treatment is the focus of the present review.

## 2. Diagnosis and Treatment of Liver Cancer

### 2.1. Imaging Diagnosis of Liver Cancer

The imaging diagnosis methods available for liver cancer mainly include ultrasound, CT imaging, MR imaging, PET/CT, PET/MRI, and SPECT. However, the above imaging techniques have both advantages and disadvantages, and each of these is applicable in specific clinical scenarios and not in others. Radiomics has emerged as a field with certain potential in the clinical diagnosis and treatment of liver cancer in recent years.

Ultrasound color Doppler flow imaging (CDFI) is the preferred imaging method among clinicians for the examination of the liver. Color ultrasound enables the screening of liver cancer lesions, determining the presence or absence of a tumor, and analysis of the nature and composition of a tumor. In addition, CDFI enables the monitoring of blood flow inside a lesion and its surroundings, obtaining the related information, etc., allowing for a qualitative diagnosis of the lesion based on the hemodynamic characteristics [15]. Contrast-enhanced ultrasonography (CEUS) enables the early detection of vascular lesions within a tumor. CEUS responds to the changes in blood vessels within a tumor based on the quantitative data on blood flow, perfusion, and related parameters in the lesion [16]. It also allows for the monitoring of changes in the microcirculation and dynamics of the tumor via combining the characteristics of ultrasound images with the enhancement curve of a lesion, and the quantitative results thus obtained are more useful in analysis and diagnosis compared to those of a subjective assessment.

Conventional CT imaging includes ordinary plain scanning and enhancement scanning examinations for exploring the basic morphological changes in a tumor, its density, the degree of tumor enhancement, etc., to estimate or evaluate the response to tumor treatment [17]. CT also enables thin layer reconstruction, such as MIP, CPR, VR, etc. CT offers a faster scanning speed, which is an advantage over magnetic resonance imaging (MRI). Therefore, CT is the most widely used imaging technique in clinical practice. The molecular targeted drugs for anti-tumor angiogenesis could affect the cell doubling time and lead to tumor necrosis; however, to date, the early detection of these changes has not been achieved, let alone the real-time detection of tumor vascular changes in lesions. CTPI (computed tomography perfusion imaging), on the other hand, allows for dynamic enhanced CT scanning. In CTPI, specific scanning time items are selected, and arterial scans are performed in multiple phases, which allows the software to analyze lesions in different periods, including the analysis of changes in tumor density, enhancement types, and curves (obtaining time density curves). The software’s post-processing workstation may also obtain multiple quantitative parameters capable of reflecting ultra-early changes in blood flow within a tumor, which occur prior to morphological changes. This is particularly beneficial in the evaluation of targeted drug efficacy. Therefore, perfusion scanning and its quantitative parameters could be applied to detect tumor angiogenesis and necrosis, monitor changes in micro-perfusion, and observe the effect of the targeted therapy based on quantitative parameters [18]. Energy spectrum CT allows for the obtention of dual-energy data through an energy spectrum analysis of the outcomes. The obtained data on the morphological changes occurring within the tissues and organs enables the achievement of material separation, extraction of proton density maps, anhydrous iodine maps, etc., and realizing optimal energy imaging. The parameter adjustment of energy levels allows for the generation of energy spectrum images of different components [19]. Certain studies have used the quantification of iodine uptake to assess the iodine content inside a tumor, which reflects changes in the levels of vascularity and perfusion inside the tumor after treatment. Energy spectrum CT imaging may, therefore, be considered an effective method for evaluating the molecular targeted therapies for liver cancer [20,21].

PET/CT, PET/MRI, and SPECT imaging offer high sensitivity. The functional imaging of tumors has a high diagnostic value and is of great significance in the application scenario of benign and malignant tumors [22,23]. However, PET/CT and PET/MRI imaging techniques are relatively expensive. SPECT is used widely, although it offers a low spatial resolution [24]. PET/CT is commonly used in the study of liver metastases [25].

MRI routine scans include both plain and enhanced scans. MRI enables both multi-sequence and multi-parameter imaging and also the non-invasive in vivo imaging of water molecules, thereby having greater diagnostic value in the clinic. Special cases also involve the clinical application of the liver-specific contrast agent gadoxetic acid disodium, which is particularly advantageous for the evaluation of benign and malignant tumors of the liver. Diffusion-weighted imaging (DWI) is an MRI technique that enables the detection of the microscopic diffusion motion of water molecules in living tissues. DWI reflects tumor activity, allows for evaluating the changes in the tumor based on the changes in tissue signal intensity, and facilitates the quantitative analysis of tumors based on the apparent diffusion coefficient (ADC) values. Using numerical features to reflect histological characteristics, DWI realizes the dual imaging of morphology and functionality. DWI and ADC maps are important tools for the early assessment of tumor therapy efficacy and response to molecular therapeutic agents [26,27,28]. Dynamic contrast-enhanced magnetic resonance imaging (DCE-MRI) and perfusion-weighted imaging (PWI) play crucial roles in the diagnosis of liver cancer. Magnetic resonance perfusion imaging is divided into drug perfusion and non-drug perfusion scanning methods. DCE-MRI enables the measurement of various parameters to reflect tumor perfusion and changes in vascular permeability, thereby assisting in the early detection of tumor vascular damage. According to the research outcomes of several mathematicians, who have nevertheless not reached any consensus so far, the treatment and evaluation effects achieved using the quantitative parameters and perfusion parameters of DCE-MRI are affirmative and are expected to play an increasingly important role in the early evaluation of efficacy [29]. MRS (magnetic resonance spectroscopy) studies have demonstrated that different tumor metabolites result in different MRI spectra, which are closely related to the tumor type and metabolic activity. Much research remains to be conducted to justify the application of MRS technology, warranting the continuous improvement of MRI software and hardware technology through efficacy evaluation.

Radiomics is an emerging field with an underlying principle of extracting certain features from an image using imaging quantitative analysis technology, which allows for the quantitative description and analysis of the morphology and pathological characteristics of lesions in the images obtained through various medical imaging methods. Radiomics has received the attention of several mathematicians as it enables the evaluation of the malignancy, grading, staging, and prognosis of tumors. Radiomics has great application prospects in tumor diagnosis and treatment, and the evaluation methods include both morphological and textural analysis. Radiomics-based evaluation has been applied to tumor chemotherapy and radiotherapy, tumor grading, and the differentiation of benign and malignant tumors, while comparative analyses of the application of genomics and radiomics are also reported [30]. Omics features and special markers have been used for the auxiliary diagnosis of lesions for which evaluation using conventional imaging methods is difficult [31]. The quantitative numerical features offered by radiomics assist in specifying the clinical diagnosis and undertaking difficult treatment decisions. Certain studies have indicated that imaging omics features have good application value for tumors (early and late stages) [32].

### 2.2. Treatment of Liver Cancer

Surgical treatment is preferred in the treatment of liver cancer. Indeed, for patients with a history of cirrhosis, even if just one liver has been affected, surgical treatment is not suitable as hepatic encephalopathy and other complications may occur after surgery. Such patients, who are not suited to surgical treatment, may be treated with other methods, such as chemotherapy, radiation therapy (RT), radiofrequency ablation, molecular targeted drug therapy, and other commonly used clinical treatment methods, together with photothermal therapy (PTT), photodynamic therapy (PDT), or a combination of multiple treatment methods that have been studied extensively in the past few years.

In the treatment of hepatocellular carcinoma of a large size, the residual volume of the liver is assessed prior to surgery, and resection is preferred if the tumor is completely removable. However, it might be difficult to completely resect the lesion in surgery in certain cases, leaving a possibility of recurrence. In such scenarios, it is necessary to plan and implement a comprehensive clinical treatment approach [33]. For instance, to destroy the tumor entirely during surgery and reduce the recurrence rate after surgery, patients might require postoperative chemotherapy or radiotherapy. Radiofrequency ablation might also be required for recurrent lesions developing after surgery [34]. The development of modern medicine seeks to minimize the trauma caused to patients while achieving the same therapeutic effect as that of traditional surgery, which has led to the emergence of minimally invasive procedures that offer the advantages of a small wound, light pain, rapid recovery, a short hospital stay, and less bleeding [35].

Chemotherapy is one of the conventional therapies used in the clinical treatment of cancer. The underlying principle may be divided into several categories [36,37,38]. The first category involves destroying the tumor cells by interfering with cell division. The second category involves interfering with the process of DNA replication and transcription, which again enables the killing of tumor cells. The administration of chemotherapy is usually conducted in accordance with the course of treatment, predisposing it to side effects arising due to multiple administrations, such as systemic toxicity and the development of drug resistance. It is clinically proven that chemotherapy prolongs the survival of patients. However, due to the non-specific nature of chemotherapeutic drugs, these drugs cannot be specifically delivered to target tissues and, therefore, exert limited therapeutic effects [39].

Radiotherapy is another commonly used anti-tumor treatment method in clinical settings. The underlying principle is the application of high-energy X-ray and gamma ray irradiation to kill tumor cells. Radiotherapy is usually performed under the guidance of CT imaging [40]. The radiation involved, however, damages the neighboring healthy tissues as well, which could lead to complications such as radioactive inflammation [41]. Nonetheless, radiation therapy for cancer has progressed significantly in recent years, although the challenge of increasing the radiation dose to the lesions while suppressing the unnecessary doses to the healthy liver tissue remains. Radiation therapy provides a non-invasive local treatment effect achieved through ionizing radiation. With time, X-ray radiation therapy has emerged, which is represented by three-dimensional conformal radiation therapy, stereotactic body radiation therapy, proton beam and particle beam therapies, which have rendered radiation therapy a safe and effective treatment option for liver cancer [42].

Gene therapy has become a research hotspot in recent years. The underlying principle of gene therapy is the targeting of exogenous therapeutic actuators into the target tumor genes, correcting them by intervening at the gene level, thereby affecting the expression of the tumor genes [43,44]. However, since nucleic acid is exogenous, an exclusion reaction might occur, and nucleic acid implantation is a genetic alteration. Therefore, the long-term consequences of gene mutation have to be investigated. Gene therapy includes various gene transfer strategies aimed at treating patients with primary and secondary liver cancer, such as gene-directed enzyme/prodrug therapy, tumor suppressor gene inhibition, tumor suppressor gene recovery, immunotherapy, anti-angiogenesis, and viral therapy.

Photodynamic therapy (PDT) is a mild local treatment method, and as an alternative treatment strategy, it has attracted much attention in the ablation treatment of superficial or lumen tumor patients. PDT belongs to the group of minimally invasive treatments for tumors using light. PDT requires the use of a photosensitizer for applying a specific wavelength laser to irradiate the tumor to be treated. Near-infrared laser is the most preferred one for this method because of its strong penetrating power. PDT offers the advantages of low systemic toxicity and no drug resistance. However, PDT may cause phototoxicity [45,46,47] due to the use of photosensitizers, which could accumulate in the body of the patient. In addition, skin damage could be a side effect as skin is sensitive to light.

Photothermal therapy (PTT) has also emerged as a research hotspot among the various therapeutic methods studies in recent years. In this therapy, the light energy generated using a near-infrared laser (NIR laser) is converted into thermal energy [48], which is directed to directly kill the tumor cells. In the entire process, no systemic damage is caused. However, this treatment method requires the use of nanoparticle materials with special photothermal property conversion functions [49]. Photothermal agents are nanomaterials capable of generating thermal energy after irradiation using a near-infrared laser. These materials exhibit strong light absorption performance and photothermal conversion. PTT involves the use of these photothermal agents to better control and select the target treatment regions and focus the thermal energy for treatment over these target tissues, thereby reducing damage to the surrounding healthy tissues. A variety of nanomaterials are used as photothermal agents, including commonly used gold nanostructures, copper mono-sulfide nanoparticles, etc. These nanoparticles exhibit strong absorption capacity in the near-infrared light region of the electromagnetic spectrum, within the wavelength range of 650–900 nm. Due to the uneven heat distribution across a tumor tissue, PTT alone might not exhibit the desired level of efficacy. Therefore, research on the synthesis of multifunctional nanocomposites that combine PTT with other treatment methods is emerging as a novel direction in the path to resolving the above-stated issues.

Cancer may be treated using several approaches. However, the efficacy of a single treatment method is relatively limited. Chemotherapy is one of the more commonly used methods for cancer treatment, although the poor targeting efficiency, easy degradation, high toxicity, and side effects of chemotherapeutic drugs, along with the extended duration of application required, could lead to tumor drug resistance. Therefore, a combination of multiple chemotherapeutic drugs might become necessary. Traditionally, when administering two chemotherapeutic agents in vivo, the drugs are injected independently, which seldom achieves the “1 + 1 = 2” effect and could even lead to an antagonistic effect. Therefore, a nanoprobe that could carry multiple drugs, which would then exhibit a superimposition effect on each other or would be released sequentially, as required, upon stimulation, has to be developed to achieve better therapeutic effects. The emergence of nanotechnology has become well integrated with the field of medicine. Nanomaterials are used in drug delivery and gene therapy [50,51,52]. While nanomaterials are applied to a wide range of scenarios in the fields of drug delivery, gene therapy, tumor imaging, etc., their application in the field of life medicine has to be intensified, and multidisciplinary cross-collaboration must be ensured.

## 3. Nano-Diagnostic Technology and Its Application for Tumor Treatment

### 3.1. Nano-Diagnostic and Therapeutic Technology

Nanotechnology is expected to play an important role in the diagnosis and treatment of cancer as it allows for the early detection of tumors and, consequently, the early understanding of the changes required in the ongoing treatment. Ralph Weissleder reviewed the progress of molecular imaging in cancer diagnosis and treatment and reported that medical imaging technology would play a central role in clinical oncology. Molecular imaging enables clinicians to locate the tumors in the patient’s body, while also revealing the expression and activity of the molecules involved in a tumor’s behavior and response to treatment [53]. The concept of precision medicine proposes that clinical cancer diagnosis and treatment require precise information, such as the location and size of a tumor and whether or not the signs of metastasis exist. However, advancements in tumor imaging have been relatively slow due to the availability of poor pharmacokinetic profiles or the high cost of clinical development of molecular imaging agents [54].

Recently, the technology community has witnessed China’s rise in the field of nanotechnology. With the increase in government funding and the improvement of research infrastructure, China has progressed significantly. Currently, China has the fastest-growing list of publications in the field of nanotechnology and also the associated industrialization to boast about. The profound impact of the development of nanotechnology, evidenced via publications in highly influential journals, has increased rapidly over the past 20 years [55].

Successful imaging agents must, therefore, exhibit better pharmacokinetic profiles and lower toxicity for clinical relevance. Over the past decades, cancer imaging studies have identified an increasing number of imaging techniques that provide anatomical and physiological information and are now used widely in clinical practice. All of these techniques require the detection of the target molecule or cell. In this context, nanotechnology could play an important role via the provision of novel imaging probes. Indeed, nano-imaging enables the earlier detection and diagnosis of cancer compared to the other existing imaging methods [52].

The progress of treatment under various pathophysiological conditions requires the development of better therapeutic agents and the use of combinations of the required therapeutic agents with integrated biomaterials. Micrometers and nanometers combined with intelligent biomaterials with sensing and response capabilities serve as important medical systems for diagnosis and treatment. Micro and nanoelectromechanical systems (MEMs and NEMs, respectively) are used widely in drug delivery, tissue engineering, etc., with significant contributions to the treatment system [56].

### 3.2. Nano-Diagnostic Agent

A nano-diagnostic agent is a nanoparticle system that combines both diagnostic and therapeutic effects in a single platform. In comparison to the other diagnostic agents, nano-diagnostic agents exhibit further advanced functions in a single platform, including sustained/controlled release, improved transport efficiency, synergistic therapy, siRNA co-delivery, etc. The exploration of efficient ways to utilize these multimodal nano-heterostructures for the development of multimodal nano-diagnostics is the focus of our research [57,58]. Table 1 presents the different nano-diagnostic platforms currently available [59].

Nanomedicine therapeutic drugs may include hydrophobic drugs, proteins, peptides, etc., as well as hydrophilic substances, and often hydrophobic and hydrophilic drugs have to be used in combination to achieve the desired therapeutic effects [60,61]. In this regard, to determine how to combine the two kinds of drugs for them to play a better role, nanocarriers could be beneficial. Nano-diagnostic agents are also applied in optical medical imaging (using fluorescent probes or quantum dots), CT, and MRI [62].

Various nano-diagnostic agents have been developed in recent years, which has led to progress in the field of nanomedicine and its clinical application, although certain challenges related to their application in vivo and in preclinical trials remain to be overcome [63]. Most studies on nano-diagnostic agents, however, have focused on the preparation, physicochemical properties, and in vitro cell culture of nano-isomers, while the research on their in vivo evaluation is limited. Among the few in vivo studies that have been conducted, either therapeutic or diagnostic effects are reported, but not both. Nano-diagnostic agents exhibit various characteristics, such as stimulus-responsive drug release (with examples of stimuli pH, temperature, magnetism, and ultrasound), synergistic effects (e.g., siRNA delivery and combination therapy), and multiple routes of administration (e.g., oral, autophagy inhibition, etc.). Iron oxide nanoparticles exhibit excellent biosafety properties as they degrade and metabolize into iron pools in the serum to either form hemoglobin or enter metabolic pathways [64]. Radiation from heavy metal quantum dots utilized for use as probes in the human body is currently a major concern.

In the future, the development and use of biocompatible, non-immunogenic, ultra-small (<5.0 nm) quantum dots for carrying nanomedicines for clearance mechanisms via renal excretion is highly anticipated. Carbon nanotubes are potentially toxic and produce oxidative free radicals in vivo, which leads to inflammation and cellular damage to organs such as the lungs and the liver via lipid peroxidation. In addition, gold nanoparticles reportedly exhibit oxidative stress-induced cytotoxicity, which, however, appears to be a common side effect observed for other nanoparticles as well [65,66].

## 4. Research Progress on Dual-Drug-Delivery Nano-Theranostic Agents for Liver Cancer

The emergence of nanomedicine and the concept of precision medicine in recent years has raised the requirements for cancer diagnosis and treatment. Therefore, it would be of great significance to realize the early diagnosis of micro-liver cancer and conduct timely and efficient treatment and efficacy evaluation to improve the survival rate of liver cancer patients. Traditionally, the evaluation of tumor efficacy was based on changes in lesion size. Advanced imaging markers were used for detecting ultra-early changes in tumor microstructure and targeting drugs to achieve early diagnosis and evaluation and accordingly provide timely guidance to clinicians, thereby improving the survival rate of cancer patients [67].

Gold nanoparticles are capable of combining different hydroxyl or carboxyl groups and utilizing their respective loading characteristics to combine multiple drugs, contrast agents, or nanoparticles, thereby conferring multiple functions to these nanoparticles. Meanwhile, the pH sensitivity and the near-infrared light characteristics of nanoparticles enable new nanoparticles to sequentially release different types of loaded drugs, thereby achieving synergistic or antagonistic effects between the drugs; this serves as a theoretical basis for the synthesis of novel drugs, targeted imaging, and treatment [29,68,69].

**Figure 1:**
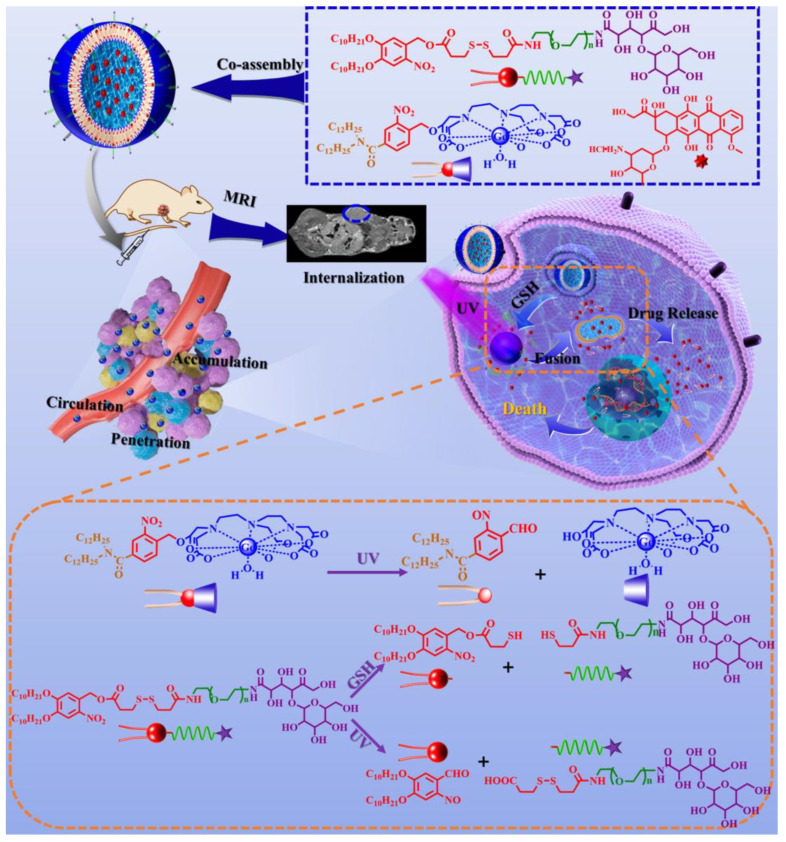
Ref. [72]. Drug delivery called the CAPIR cascade process consisting of five steps such as ① blood circulation, ② tumor section accumulation, ③ tumor penetration, ④ cell internalization, and ⑤ intracellular drug release.

Huang et al. prepared reversible disulfide crosslinked pullulan nanoparticles (FA-Pull-LA-CLNPs) with folate (FA) modification for the dual targeting and reduction-responsive delivery of anti-tumor liver drugs [70]. In addition, Huang et al. explored unique amphiphilic PCL AuNC/Fe (OH) (3)-PAA Janus nanoparticles (JNPs) to simultaneously retain hydrophilic drugs (doxorubicin) and hydrophobic drugs (docetaxel) within the different domains of the carrier system [71]. In a 2021 study by Yao et al., the GNSPLD liposomes gradually accumulated in tumor tissue after tail vein injection due to the EPR effect and targeting activity. The drug and photolysis products were released from drug-loading liposomes after internal GSH triggering and external UV triggering, which further resulted in the suppression of tumor cell growth. Treatment with GNSPLD + UV showed a better anticancer effect and fewer side effects in vivo. (Figure 1) [72]. Yan et al. used polyethylene glycol-based polymers (α-lipoic acid) and the mPEG-α-PLA copolymer to construct a double-reduction/pH response nanocarrier for capsaicin (CAP) and doxorubicin (DOX) [73]. Zeng et al. designed a dual-drug-loading nanosystem (named a THCD NP) that could selectively transport and target tumor cells for the treatment of cancer [74].

**Figure 2:**
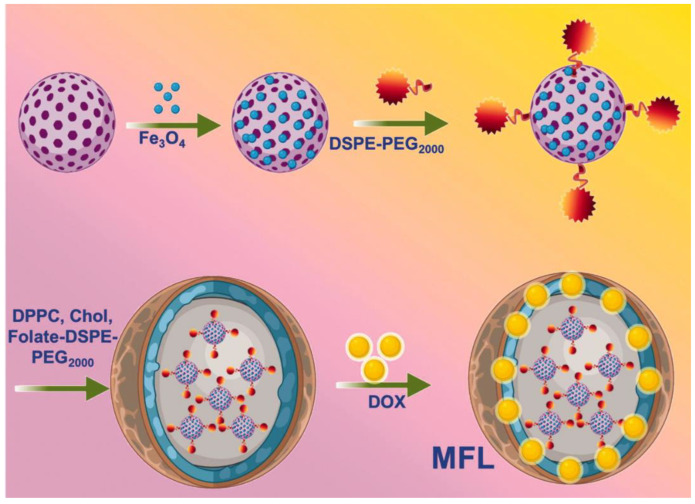
Ref. [76]. Schematic representation of the fabrication process of multifunctional liposome (MFL) drug release using the radiofrequency ablation (RF) process.

Chen et al. developed a nanocarrier based on pH/NIR dual-responsive hollow mesoporous silica nanoparticles (HMSNs) for the co-delivery of doxorubicin hydrochloride (DOX) and indocyanine green (ICG) [75]. In another experiment, researchers prepared a hybrid nanosystem using C60-Fe_3_O_4_ and functionalized it with polyethylene glycol (PEG2000), then coated it with folate receptors targeting thermosensitive liposomes and DOX. There are 80:20:5:4 ratios in optimized liposome formulations, consisting of DPPC/DSPC/DSPE-PEG2000-folate@DOX.The characteristics of the multifunctional liposome (MFL) enable them to more effectively destroy tumor cells than do non-magnetic folate-targeting liposomes. (Figure 2) [76]. Santhamoorthy et al. prepared a double-pH and thermosensitive copolymer hydrogel (HG) system (PNIPAm-co-PAAm HG) using N-isopropylacrylamide (NIPAm) and acrylamide (AAm) as comonomers [77]. Wu et al. developed a dual-targeted nanoscale drug delivery system based on EpCAM aptamers and lactic acid-modified oligoamide amine dendrimers for the simultaneous delivery of the FDA-approved drug disulfiram and the photosensitizer indocyanine green, thereby realizing a system that combined imaging and therapeutic functions in a single platform [78]. Wu et al. also prepared a dual-gated folate functionalized nanodiamond delivery system (NPFSSD), which exhibited activated fluorescence and cytotoxicity for doxorubicin [79]. Thirupathi et al. developed a dual-stimulus PNIPAm-co-PAAm-Mela/Cur HG copolymer system for temperature-responsive and pH-induced drug delivery applications. In addition, the study demonstrated that the PNIPAm-co-PAAm-Mela HG system might be used for controlled drug release to specific sites in chemotherapeutic applications [80].

**Figure 3:**
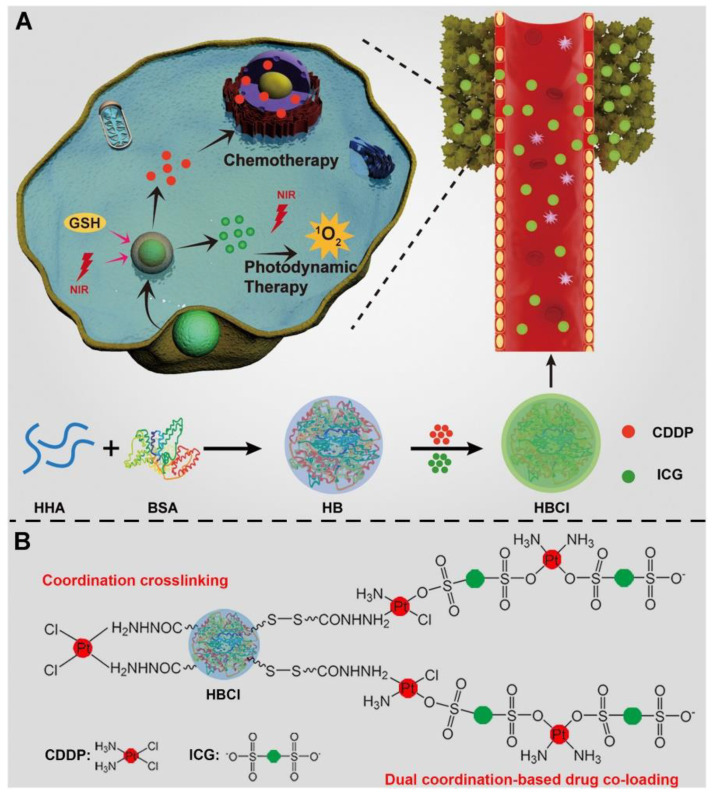
Ref. [82]. (**A**) Schematic diagram for the construction of HBCI nanoparticles and the HBCI–mediated photodynamic chemotherapy of hepatocellular carcinoma. (**B**) Schematic diagram for CDDP–crosslinking and drug loading mechanisms.

Pooresmaeil et al. designed and prepared a novel type of double-response (pH and temperature) and photoluminescence nano-gel, which was encompassed in a D-nenenebb galactose (D-Gal) moiety (CQDs/β- CD\/NIPA-M@AA-Gal). The authors then evaluated the potential of this nano-gel as a targeted drug carrier [81]. Huo et al. used the unique cisplatin hydrazide and cisplatin indocyanine green (ICG) coordination reaction and developed a multifunctional coordination nano-prodrug named cisplatin/ICG-co-loaded hydrazine hyaluronic acid/bovine serum albumin (HBCI) nanoparticles through the double coordination process of desolvation (Figure 3) [82]. Hu et al. constructed phosphate peptide-modified polydopamine-encapsulated doxorubicin-loaded hollow mesoporous organic silicon dioxide nanoparticles (pPeptide-PDA@HMONs-DOX) based on multi-modified hollow mesoporous organic silicon nanoparticles (HMONs) [83]. A study suggests that Sur@T7-AIE-Gd NPs as a novel siRNA vector nanoplatform with dual-mode imaging characteristics for the targeted and real-time monitoring of HCC which increase the accuracy and sensitivity of tumor localization and visualization [84]. Ding et al. designed and synthesized cholic acid-, galactose-, or lactose-bi-conjugated chitosan derivatives as potential anti-liver cancer drug carriers. The structures of these derivatives were characterized via proton nuclear magnetic resonance spectroscopy, element analysis, particle size distribution, zeta potential, and scanning electron microscopy [85]. Amoyav et al. explored how to maximize the therapeutic effect of the drug system by changing tissue pressure while improving drug exposure to the target organs. The authors prepared porous degradable polymer microspheres (MS), which were designed for the combined release of doxorubicin (DOX) and dexamethasone (TPZ), and introduced these drug-carrying microspheres into the liver as a hypoxic-activated prodrug [86].

**Figure 4:**
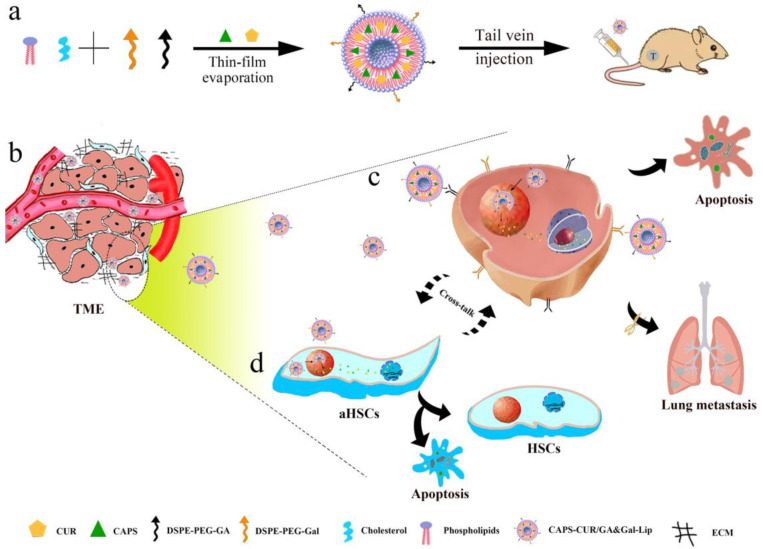
Ref. [88]. Schematic illustration of drug-loaded Lip for the inhibition of aHSC–induced drug resistance and metastasis. (**a**) Preparation of dual–ligand–modified Lip for co–delivery of CUR and CAPS. (**b**) Passive accumulation of Lip in TME. (**c**) GA–and Gal–receptor-mediated uptake and drug–-induced apoptosis. (**d**) Phenotype reversion and apoptosis of aHSCs.

Chen et al. firstly fabricated multifunctional Trojan Horse-like amphiphilic NBs; due to their unique nanostructure, they could meet the requirements for reserving hydrophobic (SF)/hydrophilic (DOX) drugs in separate rooms and releasing drugs from independent channels under pH/NIR dual-stimuli, which provides potential clinical value for preventing HCC metastasis [87]. Qi et al. used enoxolone (GA) and galactose (Gal) as target ligands to prepare novel multifunctional liposomes (CAPS-CUR/GA and Gal Lip) for the co-administration of curcumin (CUR) and capsaicin (CAPS) (Figure 4) [88]. Arafa et al. reported the design, development, and evaluation of a liver cancer-specific mitochondria-targeted double-linked metal organic scaffold (NMOF) for sequential drug delivery to cells and mitochondria [89]. Ebadi et al. discussed the physical chemistry, magnetism, and cytotoxicity of Fe_3_O_4_ nanoparticles coated with a polymer carrier and loaded with fluorouracil (5-FU)-based anticancer drugs. The synthesized Fe_3_O_4_ nanoparticles were coated with polyvinyl alcohol and Zn/Al-layered double hydroxides to serve as the main drug bodies [90]. Anirudhan et al. developed a drug delivery system based on aminated mesoporous silica nanoparticles (AMSN) and corn prolamin (a plant protein) for the co-delivery of fluorouracil (5-FU) and curcumin (CUR), and confirmed the use of prepared material as a pH-responsive dual-drug carrier to liver cancer cells [91]. Qiu et al. designed double-ligand liposomes modified with enoxolone (GA) and cyclic arginine glycine aspartic acid (cRGD) (GA/cRGD-LP) for targeting GA receptors and α-β integrin. The authors of the present review have also developed a highly selective targeted drug delivery system that could further improve the anti-tumor efficiency of drugs via the targeting of liver tumor cells and the vascular system [92].

**Figure 5:**
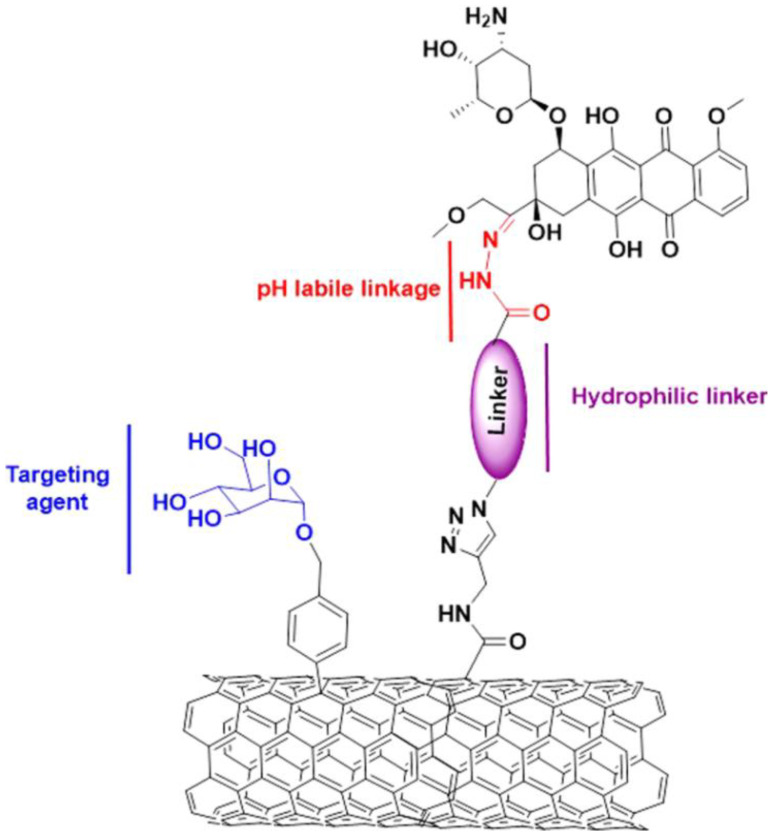
Ref. [93]. The dual functionalization of SWCNTs with doxorubicin and mannose.

Assali et al. used doxorubicin (DOX) connected with acid-labile linkage and man-nose (Man) as targeting agents for the double-covalent functionalization of single-wall carbon nanotubes (SWCNTs). The developed nano-drug was characterized using transmission electron microscopy, which revealed that the functionalized single-walled carbon nanotubes with a diameter of 6–10 nm exhibited good dispersion. In addition, the percentage of functionalization was determined through thermogravimetric analysis (Figure 5) [93]. In a study by Chen et al., they developed a novel drug delivery system of microspheres based on EC that could effectively dual-load NaHCO_3_ nanoparticles and DOX nanoparticles; microspheres loaded with NaHCO_3_ nanoparticles can continuously improve the pH of the tumor microenvironment, thereby breaking its equilibrium state and ultimately providing an enhanced effect of inhibiting the growth of tumor cells [94]. Jedrzak et al. synthesized multifunctional nanocarriers based on PAMAM dendrimer products (G) 4.0, 5.0, and 6.0 fixed on polydopamine (PDA)-coated magnetite nanoparticles (Fe_3_O_4_). The synthesized nanoplatform was characterized based on transmission electron microscopy (TEM), zeta potential, Fourier transform infrared spectroscopy (FT-IR), X-ray photoelectron spectroscopy (XPS), and magnetic resonance imaging (MRI). In addition, as a proof of concept, it was confirmed that the G5.0 functionalized nanocarrier could be successfully applied to achieve the combined chemotherapy and photothermal therapy (CT-PTT) of liver cancer cells [95]. Pandey et al. designed and synthesized amphiphilic biocompatible mikto-arm star copolymers comprising a two-hydrophobic-polymer-(ε-caprolactone)-based block, a short poly-(alkynylglycine) intermediate block, and a hydrophilic galactose peptide block. The star-shaped copolymers were initially self-assembled into uncrosslinked (UCL) micelles, with the free alkyne groups on the core–shell interface of the UCL micelles, and then crosslinked with bis-(azidoethyl)-disulfide (BADS) through click chemistry to form interface-crosslinked (ICL) micelles [96]. Yang et al. prepared bifunctional albumin-based nanoparticles (gal-BSA NPs) using sonochemical methods and reported that these NPs could effectively encapsulate bilirubin (BR) owing to their adsorption ability and hydrophobic interactions. Moreover, the possibility of blank gal-BSA NPs replacing BSA and having a better adsorption capacity for excess BR was also stated. In addition, the potential anti-tumor activity of BR against HepG2 cells was explored and GSH-reactive NPs loaded with gal-BSA were developed for the treatment of cancer [97].

**Figure 6:**
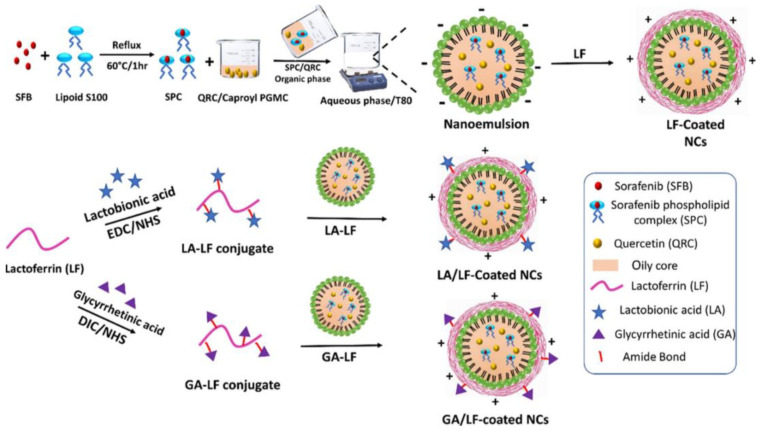
Ref. [100]. Schematic diagram for the preparation process of LF–coated NCs.

Wu Hao et al. constructed pH/enzyme-responsive polymer prodrug nanoparticles with a 10-HCPT structure. At pH 7.4, the nanoparticles exhibited a core–shell structure and good in vitro stability with little drug release. However, when exposed to pH 5.0, the nanoparticles exhibited a nanogel-like morphology, and in the presence of 2 μM papain, the cumulative drug release rate was as high as 71.4% in 60 h, which was almost twice as high as that in the case of 0.2 μM papain, suggesting that dual stimulation by both the enzyme and pH can significantly enhance selective drug release from tumor cells [98]. Zhang et al. modified our previously developed catadine-loaded BR2 liposomes with anti-CA IX antibodies, which improved their targeted delivery of drugs to cancer cells through the highly expressed carbonic anhydrase IX (CA IX) receptor. The cellular uptake of bifunctional liposomes (DF-Lp) was observed to be higher than that of the other treatments. The induction of CA IX overexpression led to the higher cell binding of DF-Lp, resulting in an excessive antibody blockade that led to a decrease in cancer cell correlation, indicating that the liposomes exhibited specific targeting of the CAIX-expressing cells [99]. In a study, Abdelmoneem et al. were inspired by the targeting action of lactoferrin (LF) via binding to LF receptors overexpressed by HCC cells, and lactoferrin shell-coated oily core nanocapsules (LF-NCs), glycyrrhetinic acid (GA)-targeted and lactobionic acid (LA)-targeted LF-NCs were fabricated for the combined delivery of hydrophobic drugs, sorafenib (SFB) and quercetin (QRC), and they proposed a potential therapeutic HCC strategy. (Figure 6) [100]. Singh et al. developed a dual-drug delivery platform named “Biphasic”, which could target cancer cells and also eliminate bacteria in the ecological niche of cancer. Binuclear liposomes include liposomes loaded with anticancer drugs (such as doxorubicin) within their cores and lipids loaded with cationic Cathelicidin (sushi S3) on their surfaces. Folic acid also adheres to the surface of liposomes, conferring cancer cell specificity [101].

Bullo et al. designed graphite oxide polyethylene glycol (GOPEG) nanocarriers and loaded them with two anticancer drugs, protocatechuic acid (PCA) and chlorogenic acid (CA). The designed anticancer nanocomposite was then encapsulated inside folic acid to facilitate cancer cell targeting as folic acid receptors were overexpressed on its surface [102]. Luo et al. developed a dual-pH/redox-responsive hybrid PM through the self-assembly of two amphiphilic diblock copolymers named polyethylene glycol methyl ether poly-(-amino ester) (mPEG-b-PAE) and polyethylene glycol methyl ether grafted disulfide poly-(amino ester) (PAE-ss-mPEG) for the delivery and controlled release of anticancer drugs [103]. Ni et al. synthesized the biotin/lactic acid-modified polyethylene glycol lactic acid polyethylene glycol (BLPP) copolymer and curcumin- and fluorouracil-loaded nanoparticles (BLPPNPs/C+F) to achieve the enhanced treatment of hepatocellular carcinoma. The in vitro and in vivo studies demonstrated that the blank BLPPNPs exhibited good biocompatibility [104]. Zhong et al. proposed a novel multi-component microsphere (MCM) system with co-encapsulation and spatiotemporal drug release capabilities for the postoperative treatment of cancer and liver regeneration, and this system could be loaded with doxorubicin (DOX) and liver regeneration-enhancing factor (ALR) into its shell and core, respectively. In addition, these MCMs exhibited a rapid release of DOX and a persistent release of ALR. These dual-drug-loaded MCMs exhibited significant postoperative tumor-killing effects and the promotion of liver regeneration [105]. Espinoza et al. studied the efficiency of a dual-pH-sensitive intelligent nanocarrier based on silica nanoparticles (SNPs) extracted from rice husk ash (RHAs) in the inhibition of liver cancer cell proliferation. The SNPs were coated with chitosan (CH) and then loaded with doxorubicin (DOX), followed by functionalization with the cell membrane (CM) to realize the homologous targeting ability [106].

The emergence of a series of nano-diagnostic and therapeutic platforms, modified using different methods, has enabled the realization of various functions, such as targeted osteoporosis, photothermal combined chemotherapy, the integration of imaging and treatment, etc. Meanwhile, as there is a possibility of these agents having an amphiphilic nature, the current research could attempt to load targeted molecules and fluorescence imaging substances on these agents and conduct experimental studies on other types of tumors using these agents.

## 5. Conclusions and Outlook

At present, there are still some problems to be solved in nano research; the different pharmacokinetic characteristics of different drugs put forward different requirements for nano-drug-carrying synthesis, which further increases the difficulty of drug-carrying system research and development. Nano-drugs have potential safety issues and toxicity risks, and drugs will be gradually released during the circulation process, causing systemic toxicity and reducing the efficiency of tumor delivery. How to optimize the drug loading rate of nano-drugs, reduce and solve the toxicity of drug loading, and better achieve clinical translation are important challenges we are facing.

Nano research should be carried out in collaboration with clinicians, especially in the case of the accumulation of clinical research data, to carry out innovative research that meets clinical needs and realizes real clinical value. It should apply the translational field not only to more traditional liposomes, but also to polymer micelles, and other such primary level applications toward functional, targeted, and environmentally sensitive nanomedicines. Complex toxicity assessment procedures are required for newly developed nanomaterials. Designing and developing new models of cross-fertilization of AI with nanomedicines is also a hotspot for future research to further improve their anti-tumor effects and facilitate their clinical translation. The use of nanocarrier drug therapy offers the possibility of treating a number of diseases that have long been considered untreatable. dual-drug-delivery nano-theranostic Agents provide new ideas for the bioavailability of drugs and shorten the treatment time. It is reasonable to believe that nanotechnology will make an important contribution to tumor control.

Nano-diagnostic and therapeutic agents modified using different methods are expected to achieve the early and specific diagnosis of liver cancer and detection of changes in lesions. Therefore, nano-level diagnosis and treatment could become the focus of medical research in the future.

## Figures and Tables

**Table 1:** Ref. [59]. The current advanced theranostic nanomedicine platforms for the integration of diagnosis and therapy.

Type of Theranostic Nanomedicine	Material (s)	Therapeutic Agent	Diagnostic Agent	Size	Targeting Agent	Advancement
Drug-polymer conjugates	HPMA	64Cu	64Cu	N.A.	RGD	Cancer imaging and radiochemo-therapy
Polymeric nanoparticles	PLA-TPGS	Docetaxel	Quantum dots	~250 nm	Folic acid	Co-delivery of docetaxel and quantum dots
Solid lipid nanoparticles	Low-density lipoprotein, Cholesterol	Paclitaxel/ siRNA	Quantum dots	~130 nm	cRGD	Multimodal therapy
Dendrimers	Polypropyleni-mine	Phthalocyanines	Phthalo cyanines	~62 nm	LHRH	Delivery of single theranostic agent
Liposomes	TPGS, Phospholipids, Cholesterol	Docetaxel	Quantum dots	~210 nm	Folic acid	Co-delivery of docetaxel and quantum dots
Micelles	TPGS	Iron oxide nanoparticles	Iron oxide nanoparticles	~178 nm	Passive	Delivery of single theranostic agent
Gold nanoparticles	Gold nanoparticles	DOX	Gold nanoparticles	~55 nm	CPLGLAGG peptide	Stimulus responsive drug release
Carbon nanomaterials	SWCNTs	Intrinsic property	Intrinsic property	Length of ~140 nm	Passive	Self-photoluminescent and photothermal property

## Data Availability

Not applicable.
